# Modification of bio-based β-diketone from wheat straw wax: synthesis of polydentate lipophilic super-chelators for enhanced metal recovery†

**DOI:** 10.1039/c8ra09426h

**Published:** 2019-01-25

**Authors:** Kaana Asemave, Fergal P. Byrne, James H. Clark, Thomas J. Farmer, Andrew J. Hunt

**Affiliations:** Green Chemistry Centre of Excellence, University of York York YO10 5DD UK thomas.farmer@york.ac.uk; Chemistry Department, Benue State University Makurdi Nigeria; Materials Chemistry Research Center, Department of Chemistry and Center of Excellence for Innovation in Chemistry, Faculty of Science, Khon Kaen University Khon Kaen 40002 Thailand andrew@KKU.ac.th

## Abstract

Bio-derived lipophilic polydentate chelators have been synthesized and tested for their chelating ability using a range of metal salts of Cu, Co, Ni, Fe, and Cr. These novel molecules were produced by the Michael addition reaction of 14,16-hentriacontanedione, isolated from wheat straw wax, with methyl acrylate or bio-derived dimethyl itaconate *via* microwave heating. The Michael adducts could either be used directly as esters or be hydrolysed to their acid form. Critically, the creation of additional binding sites *via* the carboxylate moieties leads to an enhanced metal uptake over both a non-renewable commercially available lipophilic β-diketone (dibenzoylmethane) and the unmodified hentriacontane-14,16-dione, for the chelation of Fe(iii), Cr(iii) and Ni(ii). The modified β-diketone containing a single carboxylic acid functionality was able to extract 167 mg L^−1^ of Fe(iii) from an FeCl_3_ solution with no pH adjustment. In comparison, no chelation was observed with dibenzoylmethane, while unmodified hentriacontane-14,16-dione was able to extract 81 mg L^−1^. The modified chelators containing one and two ester carboxylates extracted 255 and 305 mg L^−1^ Cr(iii) from a solution of CrCl_3_ at pH 5–6, 238 mg L^−1^ was extracted by the unmodified β-diketone whilst no extraction was observed using dibenzoylmethane. This suggest some minor contribution or positive effect to chelation due to neighbouring ester groups. The chelator containing two carboxylic acid groups (tetra-dentate when combined with the diketone) was the most proficient in this study for removal of Ni from an NiCl_2_ solution (140 mg L^−1^). It was also found that at higher pH almost quantitative extraction was achieved using the polydentate chelators.

## Abstract 2

Bio-derived lipophilic polydentate chelators have been synthesized and tested for their chelating ability using a range of metal salts of Cu, Co, Ni, Fe, and Cr.

## Graphical abstract

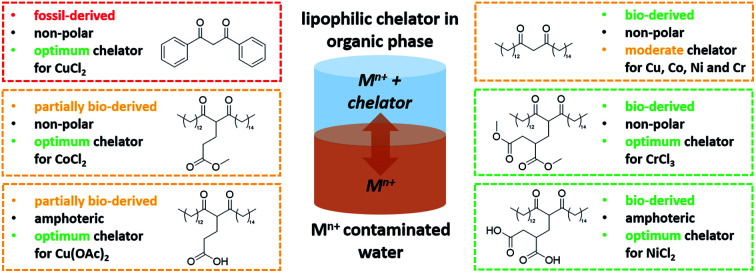

## Introduction

**Fig. 1:**
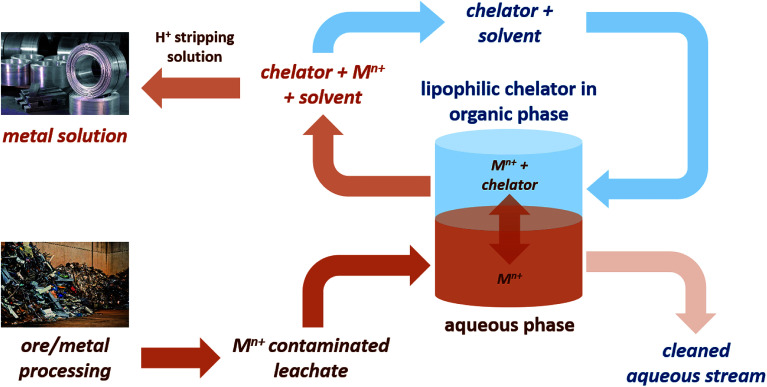
Application of a bi-phasic metal recovery system using lipophilic bio-based chelators.

Concerns over the security of critical element supplies that are vital to the chemical industry have created the need to find new sources of these elements. Recovery from aqueous waste streams provides a method of isolation of dispersed metals, some of which are toxic to the environment, for which chelating agents are a useful tool.^1,2^ However, many traditionally used chelators are often water soluble, petroleum-derived and suffer from high toxicity, persistence and/or bioaccumulation.^3,4^ Lipophilic chelators are of significant interest as they allow for application of a biphasic metal recovery system (Fig. 1), and indeed some lipophilic petroleum-derived chelators do exist, including dibenzoylmethane, 1.

**Fig. 2:**
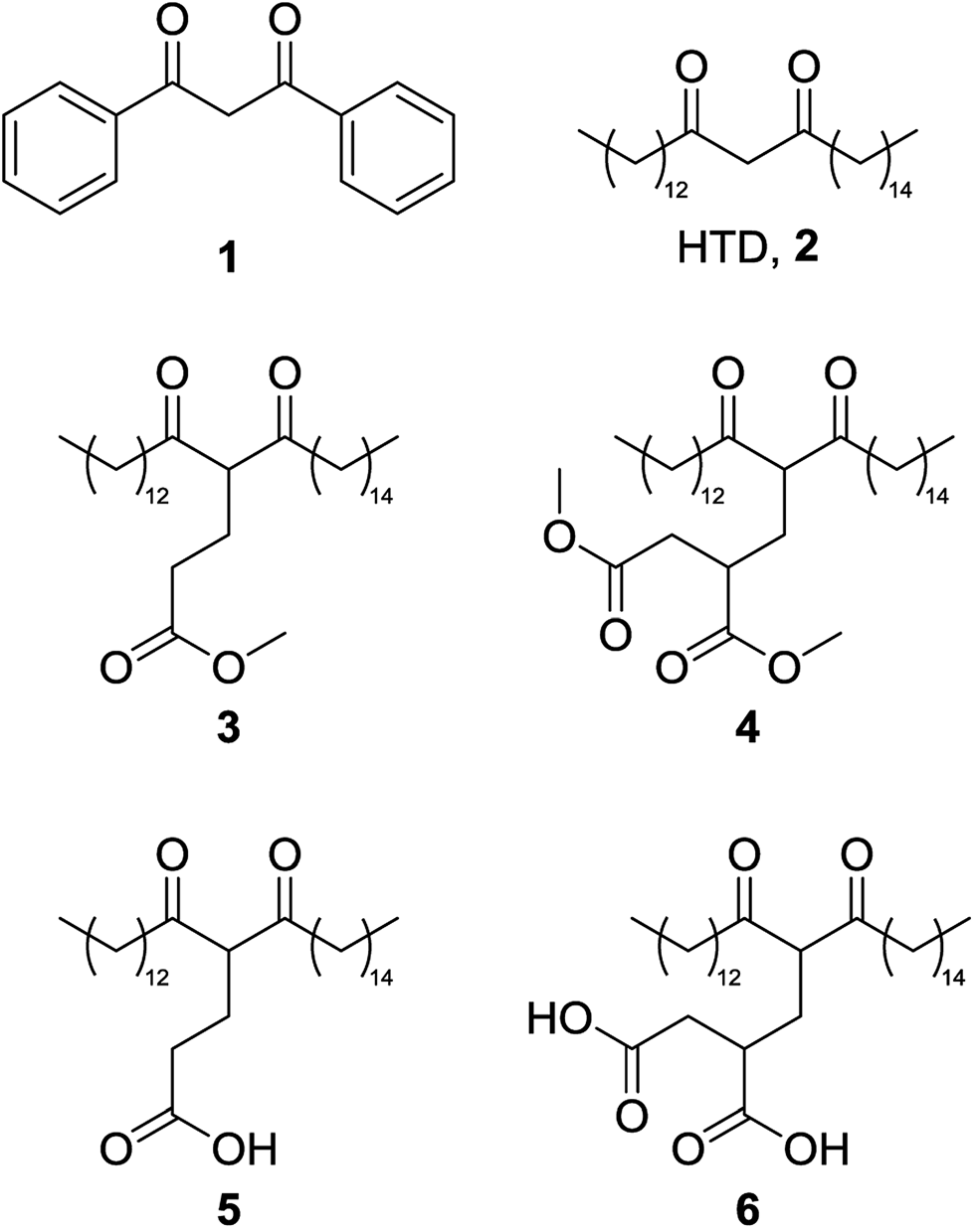
Dibenzoylmethane, 1; hentriacontane-14,16-dione, 2; the ester forms of the Michael addition reaction products from methyl acrylate, 3, and dimethyl itaconate, 4; and the carboxylic acid forms of the Michael addition reaction products from methyl acrylate, 5; and dimethyl itaconate, 6.

The development of bio-derived lipophilic chelators from agricultural wastes has the potential to contribute to a holistic metal recovery system as part of a circular economy. One such chelator, hentriacontane-14,16-dione (HTD), 2, a major component of wheat straw wax, has recently been reported as an effective bio-derived chelating agent (Fig. 2).^5^2 is a lipophilic β-diketone that can be extracted from the raw biomass using green solvents such as supercritical carbon dioxide.^6^ As it is composed only of carbon, hydrogen and oxygen, the release of nitrogen or phosphorous into the environment can be avoided, and its high log *P*_(o/w)_ means efficient extraction from the aqueous phase can be achieved.^5^ In addition, being extracted from a waste agricultural residues such as wheat straw wax means an abundant supply is ensured.^5^

In this current study, it was hypothesised that further modification of 2 with carboxylate groups has the potential to improve chelation ability. For example, hydrolysis of the product of the Michael addition reaction between α,β-unsaturated carbonyl esters and β-diketones could provide such functionality to enhance the chelation ability of the β-diketone. Due to the presence of lipophilic alkyl chains and polar carboxylic acid groups, these modified β-diketones can also be applied as surface active agents, as previously suggested by Fanou *et al.*^7^

Importantly, the Michael addition is a favourable reaction from a green chemistry perspective due to its 100% atom economy.^8^ The Michael addition has previously been shown to be suitable for the use of microwave heating, which increases its greenness even further due to improved energy efficiency compared to conventional heating.^9^

Although Michael addition reactions are often carried out in polar solvents such as MeOH, acetone, DMSO, THF,^10^ MeCN,^11^ Ravichandran and Karthikeyan^12^ have previously reported solvent-free Michael addition reactions. There are several advantages to this. Waste, hazards and toxicity associated with many organic solvents are eliminated and the risk of pressure build up is reduced, thus easing scale-up.^9,13^ The combination of both solvent-free, microwave-heated Michael addition reactions has also been performed by Rao and Jothilingam.^14^ It was found that solvent-free condition showed improved reaction rates compared to when polar aprotic solvents, such as DMSO, were used.^14^

Several heterogeneous bases have been found to be excellent catalysts for promoting Michael addition reactions.^15,16^ KF/alumina in particular has attracted widespread interest due to the simplicity of its use and environmentally-benign production.^17,18^ It has been shown to be effective at a low loading of 5 mmol g^−1^ KF on alumina in the reaction of acetylacetone with methyl acrylate.^19^

This protocol was utilised by Farmer *et al.* for the Michael addition of acetylacetone to the bio-derived α,β-unsaturated diesters, dimethyl itaconate (DMI) and dimethyl fumarate.^19^ However if was never studied if “super-chelators” with an enhanced ability to chelate ions were obtainable *via* hydrolysis of the esters, this would have yield two acid groups to accompany the chelating diketone. However, these super-chelators would likely be water soluble, so extraction from an aqueous phase would be impossible. The above system was however extended further with the addition of acetylacetone onto bio-based unsaturated polyesters, and although these diketone pendant polymers were shown to chelate Fe(iii) they were too polar (ethanol soluble) for use in a biphasic recovery system.^20^

Herein, lipophilic bio-derived bifunctional “super-chelators” with enhanced chelation-ability as compared to commercially available lipophilic β-diketone have been synthesized and performance-tested using a range of metal salts. The super-chelators were produced by the Michael addition reaction of 2 with the bio-platform molecules methyl acrylate and dimethyl itaconate, to yield chelators 3, 4, 5 and 6 (Fig. 2). KF/alumina was employed as a heterogeneous base catalyst under microwave irradiation in a solvent-free system, two factors which further accentuate the green credentials of the process. These lipophilic keto esters (3 and 4) can be used as chelators directly or they can be hydrolysed to their keto acid forms which could potentially enhance chelation-ability. The hydrolysis products, 5 and 6, have both β-diketone functionality as well as two acid groups for enhanced chelation ability. The chelating abilities of 3, 4, 5 and 6 were tested using a range of metals salts in comparison with 2 and the traditional petrochemical-derived chelator, dibenzoylmethane, 1.

## Experimental section

A summary of the methods used are given below, further Experimental detail (materials and equipment (Section S1.1.), characterisation of isolated compounds, detailed procedure of extraction of metal ions) is presented in ESI.†

### Purification of hentriacontane-14,16-dione from wheat straw wax

The isolation of the hentriacontane-14,16-dione (HTD) was carried out as previously reported by Horn *et al.*^21^ with some slight modifications. A typical extraction procedure and accompanying compound characterisation is presented in the ESI (Section S1.2.).†

### Preparation of the KF/alumina

KF/alumina was prepared as previously reported by Farmer *et al.*^19^ and Lenardão *et al.*^17^ The complete procedure is presented in the ESI (Section S1.3.).†

### Preparation of trimethyl aconitate

Aconitic acid (5 g, 0.029 mol) and methanol (33.3 mL, 0.823 mol, 30 molar equivalents) were placed into 250 mL round bottom flask along with 3 drops of concentrated sulphuric acid. The reaction mixture was refluxed overnight with continuous stirring. After 18 h, the methanol was removed and fresh methanol (33.3 mL, 0.823 mol) and 3 more drops of sulfuric acid were added. The system was refluxed for another 18 h. After the reaction, the reaction mixture was transferred to a separating funnel along with water (75 mL). The reaction vessel was rinsed with petroleum ether (60–80 °C). After shaking, settling and separating, the organic layer was washed with 15 mL of distilled water, followed by 25 mL of 5% sodium bicarbonate, followed by 15 mL brine solution, at which point it was dried with anhydrous magnesium sulphate and filtered. The solvent was removed *in vacuo* to give the product, a pale-yellow oil. The yield obtained was 85%. The full characterisation of the resulting molecule is presented in the ESI (Section S1.4.).†

### Modification of the bio-derived β-diketone

Based on the method previously reported by Asemave *et al.*^22^ Into a 15 mL microwave vial, 14,16-hentriacontanedione (HTD) (0.010 g, 0.02 mmol) was added; followed by a known amount of KF/alumina. The heterogeneous catalyst was well dispersed among the HTD. Dimethyl itaconate (0.014 g, 0.09 mmol, 4.5 mole equivalents) or methyl acrylate (10 μL, 0.11 mmol, 5.5 mole equivalents) was then added to the reaction mixture for the synthesis of 3 and 4 respectively. The maximum pressure and the power in the CEM discover microwave was set at 300 psi and 300 watts respectively at a known reaction time and temperature. After the reaction, the reaction mixture was filtered to remove the catalyst using dichloromethane and ethyl acetate. The products were purified using flash chromatography. In addition, short part distillation (Kugelrohr) was also applied to separate the excess dimethyl itaconate from the modified HTD.

When the reaction was conducted with conventional heat source (stirrer hot plate), identical quantities of materials were added to a 15 mL vial with screw cap. The reaction mixture was well stirred during the reaction time under solvent-less conditions. The reactions were also scaled up using 0.2 g HTD, 1 g KF/alumina with 4.5 mole equivalents of the dimethyl itaconate or 5.5 mole equivalents of the methyl acrylate. The full characterisations of resulting molecules are presented in the ESI (Section S1.5.).†

### Hydrolysis of the modified β-diketone

3 (0.0692 g) and 4 (0.0479 g) were dissolved separately in dichloromethane in 50 mL vials. Into these solutions was added a saturated solution of sodium hydroxide in methanol. The ratio of dichloromethane : sodium hydroxide solution was 1.5 : 1. The two mixtures were stirred overnight at 30 °C. The mixtures were dark yellow at the beginning of the reaction and became white and cloudy over time, indicating formation of the Na salts. The reactions were stopped, and the solvent removed. The solid residues were then dissolved separately in 10 mL water and acidified with hydrochloric acid to a pH of 1–2, in order to obtain the carboxylic acid forms of the products (5 and 6). The mixtures were then extracted using dichloromethane. The full characterisations of resulting molecules are presented in the ESI (Section S1.6.).†

### Procedure of the metal ions extractions

An equal volume (5 mL) of an aqueous metal solution of known molarity was combined with a cyclohexane/chelator solution of known molarity in a 50 mL vial with screw cap at 20 °C and vigorously agitated for 30 min, as previously reported in the literature.^7,23,24^ Thereafter, the sample was allowed to stand for 24 h. A blank (control) sample containing an aqueous metal solution and cyclohexane only was treated as described above to determine the distribution ratio, *D*. The aqueous phase was carefully removed and the absorbance of the residual metal ions in the aqueous solution was measured by UV/Visible spectrophotometer. The concentration of metal in the raffinates and blanks were measured by carefully removing the aqueous phase from the mixtures and measuring the absorbance of the residual metal ions by UV/Visible spectrophotometer. The UV/Visible spectrophotometer was calibrated using aqueous metal solutions of varying concentrations. The actual amount of extracted metal was determined by multiplying the observed amount by the distribution ratio, *D*.^25,26^ The pH of the aqueous phase after extraction was recorded as the equilibrium pH. The pH was adjusted with sodium hydroxide or hydrochloric acid in some cases as specified. Full details for the change of pH for each metal salt is presented in the ESI (Section S1.7.).†

## Results and discussion

### Synthesis of super-chelators

**Fig. 3:**
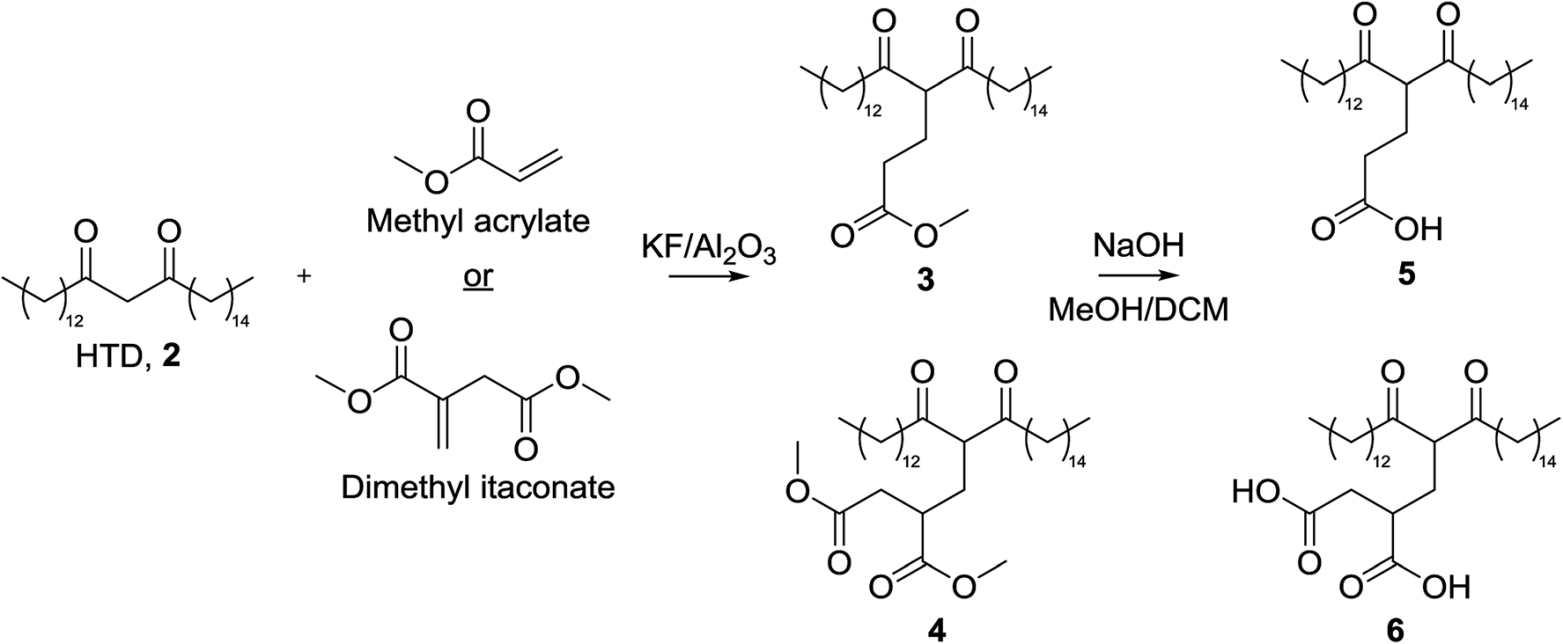
Reaction scheme for the Michael addition of methyl acrylate (MA) or dimethyl itaconate (DMI) onto HTD, 2, this used in the synthesis of 3 and 4. Subsequent hydrolysis was used for the synthesis of 5 and 6.

The low p*K*_a_ of the carboxylic acid protons of the Michael acceptors meant they would preferentially deprotonate in the presence of a base catalyst. As such, the ester form of the Michael acceptor was used in the reaction (Fig. 3), and a more detailed discussion following our first report of this modification is contained herein.^22^ Initially, methyl acrylate (MA), methyl methacrylate (MMA), dimethyl itaconate (DMI) and trimethyl aconitate (TMA) were included as Michael acceptors, but the low yields obtained by MMA and TMA meant they were not included for the remainder of this investigation. It is thought that the steric hindrance on TMA prevented the reaction from occurring, and electron-donation from the methyl group to the alkene of MMA reduced its electrophilicity.

Based on our previous study into modification of itaconates we chose to use KF/alumina as catalyst with a loading of 5 mmol of KF per 1 g of alumina for all screening reactions, this having adequate basicity for the reaction whilst maintaining a reasonable surface area (30 m^2^ g^−1^).^19^

**Fig. 4:**
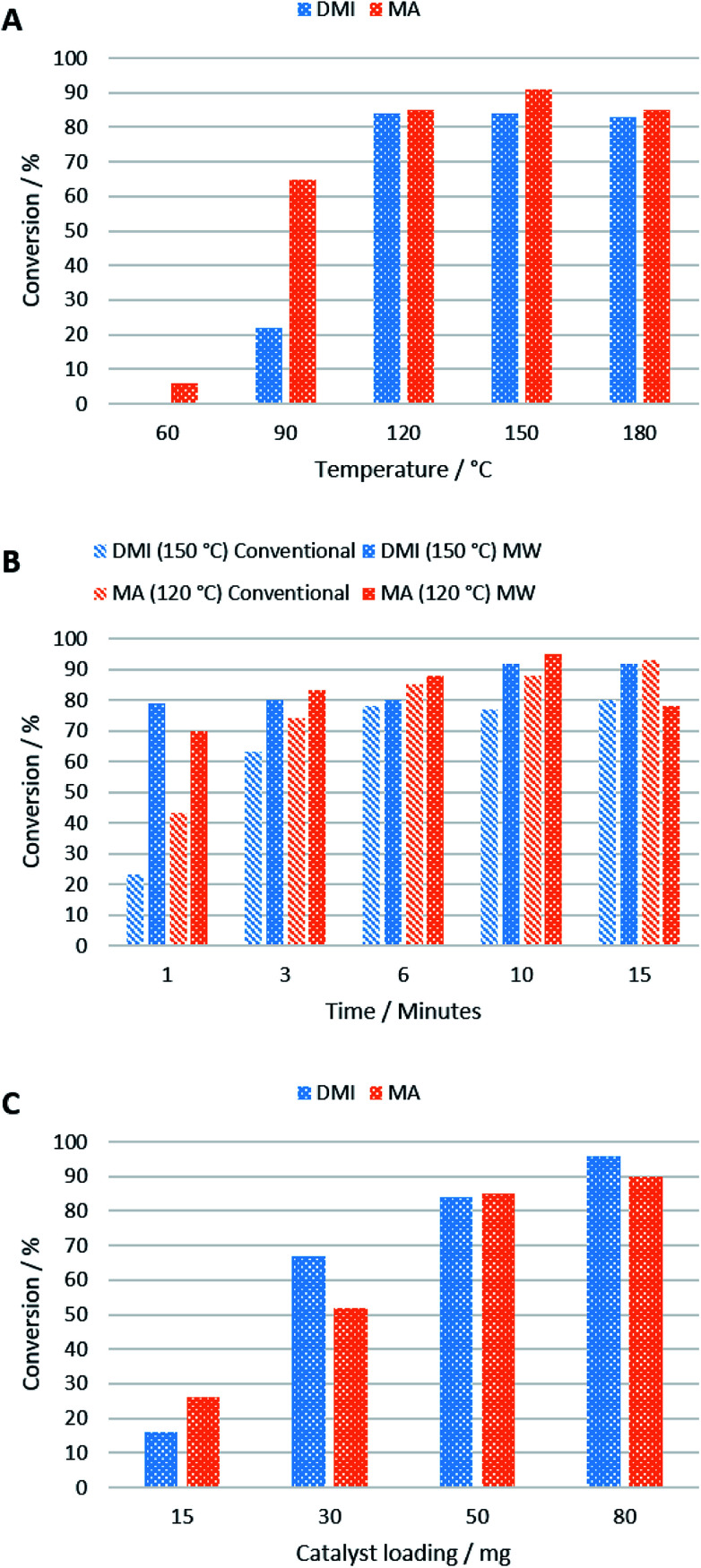
Conversions of DMI and MA in the Michael addition reaction with 2 under different conditions in pressure vessels. (A) Optimisation of temperature when 50 mg of catalyst was used with a reaction time of 5 min; (B) optimisation of time using 50 mg of catalyst at 120 °C (MA) or 150 °C (DMI); (C) optimisation of catalyst loading at 120 °C (DMI) or 60 °C (MA) after 5 min.

A temperature screen showed that 120 °C was optimal for the reaction of MA and DMI with 2 when using 50 mg of catalyst after 5 min, above which no improvement in conversion was observed (Fig. 4A). This is perhaps due to surface water on the KF/alumina being driven off, improving its catalytic activity. Yields of 84% and 85% were obtained for DMI and MA respectively at this temperature after 10 min, after which no improvement in yield was observed. The reaction with MA still occurs even at lower temperatures, in good agreement with previous studies,^27^ but above the optimal temperature of 120 °C, side reactions increased. It was also found that DMI isomerised to citraconate at 90 °C and above in the presence of the KF/alumina which could negatively affect the conversion of 2.

Microwave and conventional heating displayed similar performances in terms of reaction rate, with microwave heated reactions achieving higher conversions after 6 min and 150 °C, using 50 mg of catalyst (Fig. 4B). Due to the high polarity of carbonyl groups, ketones readily absorb microwaves and can be heated efficiently by microwave irradiation.^28^ As expected, the reaction time is slower when using 2 as Michael donor compared to acetylacetone, as reported by Farmer *et al.*,^19^ due its higher viscosity and lipophilicity. The reason microwave heating only performed marginally better than conventional heating in this work is likely due to the small scale at which the reaction was performed. On a large-scale, conventionally-heated batch reactor, heat transfer would inhibit the reaction rate significantly compared to a microwave heated reactor, which can permeate larger volumes more efficiently.^29^

The reaction rate was found to increase with increasing catalyst loading from 15 mg > 30 mg > 50 mg with only a slight improvement obtained when 80 mg catalyst was used to convert 10 mg of 2 after 10 min at 120 °C (DMI) and 60 °C (MA) (Fig. 4C). Although the optimal catalyst loading is large (500%), the catalyst is heterogeneous and highly recoverable, so it would be suitable for use in a continuous flow reactor where this is a non-issue. Yields of 90% and 96% were obtained for 3 and 4 respectively at the optimal temperature.

MA has been previously reported to undergo double addition to Michael donors, and the same effect occurred with 2. As the first α-proton is much more acidic than the second α-proton, the strength of KF/alumina as a base is demonstrated. Purification of the double- and single-addition products of MA was achieved by column chromatography. Only the single addition product was used for subsequent chelation testing. Double addition did not occur with DMI due to steric hindrance.

In summary, optimal conditions were 50 mg of KF/alumina (500% loading relative to the β-diketone) at 120 °C for 10 min at which point the recoverable yield of 3 and 4 was 60% (41% double addition product and 19% single addition product) and 20% respectively. Further to our earlier modification, hydrolysis of the esters 3 and 4 to form the corresponding carboxylate salt was carried out using sodium hydroxide, the aim of which was to generate chelators carrying multiple negative charges. Methanol is known to enhance the hydrolysis of esters due to its high polarity. However, its polarity was too high to dissolve 3 and 4. Therefore, methanol was used in combination with a co-solvent of lower polarity. A 60 : 40 dichloromethane/methanol solvent system was used, in which the sodium carboxylate salt was fully formed after 24 h. Treatment with dilute hydrochloric acid yielded the free acids 5 and 6 (83% yield in both cases).

### Chelation tests with super-chelators in comparison with a traditional chelator, dibenzoylmethane

The modified ester (3 and 4) and carboxylate (5 and 6) chelators were tested for their chelation ability in comparison with unmodified HTD, 2, and a traditional fossil-derived chelator, dibenzoylmethane, 1. It was previously reported that initial concentration of metal ions affects the efficiency of extraction.^30^ Hence, two significantly different metal/ligand (M/L) ratios were first tested (10 : 1 and 2 : 1) and, as expected, higher metal/ligand ratios led to more metal extraction at an unadjusted pH of 4–5 after 30 min (Table 1).

**Table 1:** The effect of metal/ligand ratio and pH on the extraction of CuCl_2_ using each chelator

Chelator	Amount of metal extraction/mg L^−1^		
	M/L ratio = 2 : 1, pH = 5.56	M/L ratio = 10 : 1, pH = 5.56	M/L ratio = 10 : 1, pH 6.18
1	25	120	224
2	19	108	529
3	6	107	412
4	9	102	519
5	6	88	489
6	10	86	494

Higher pH is also known to enhance metal extraction ability of β-diketones.^30^ The pH of 5.56 was selected and corresponds to the value for the CuCl_2_ solution without any adjustment. A second set of extraction tests were conducted at pH = 6.18. In all cases, particularly with the bio-derived chelators, the amount of Cu(ii) was significantly enhanced at pH = 6.18 (Table 1). It was not possible to increase the pH further due to precipitation of metal salts.

**Fig. 5:**
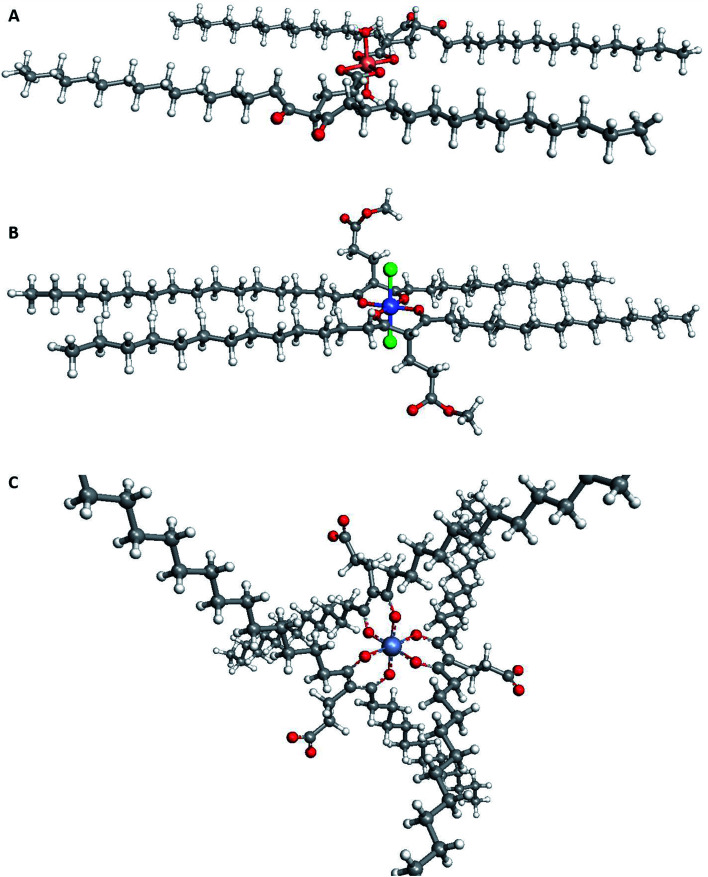
Suggested structures for extracted complexes of different metals. (A) [Cu(5)_2_OH_2_] as extracted from CuOAc_2_; (B) [Co(5)_2_Cl_2_] as extracted from CoCl_2_; and (C) the surfactant [Cr(5)_3_]. As the chelators were waxes, it was not possible to obtain crystal structures. Instead, ArgusLab^32^ was used to generate images of the optimised geometry of each chelator.

Using copper as the target metal, the effect on extraction ability in each chelator was investigated using a range of copper salts, as shown in Table 2. No adjustment of pH was made, and the pH of each solution before and after extraction can be seen in Table S1 (ESI Section S1.7.).† It was found that OAc^−^ is a strong inner-sphere ligand and was useful for the highlighting the benefits of the new multifunctional chelators. As 5 and 6 also contained relatively low p*K*_a_ carboxylate functionality as well as diketone functionality, they could compete with the OAc^−^ ligand for the metal centre and were able to extract the most Cu(ii) from the aqueous phase (232 and 226 mg L^−1^ respectively). Due to the highly conjugated structure of 1, its low p*K*_a_ allowed it to form a charged species and compete with OAc^−^ for Cu(ii), albeit to a lesser extent (118 mg L^−1^). In contrast, the less conjugated (and hence assumed higher p*K*_a_) diketone groups on 2, 3 and 4 meant that the formation of a charged species was less likely. Significantly, the acids 5 and 6 were the only chelators able to remove Cu(ii) from Cu(NO_3_)_2_ (28 and 78 mg L^−1^ respectively) and from CuSO_4_ (15 and 16 mg L^−1^ respectively), demonstrating their enhanced chelating ability. Cl^−^ was also an effective counter ion for the uptake of Cu(ii) by all chelators. This is perhaps due to it being a strong inner-sphere ligand which would allow neutral diketone moieties to chelate to the metal centre and extract it into the organic phase as an octahedral complex, this predicted using ArgusLab as presented in Fig. 5A.^30,31^ It should be noted however that 1 was the best chelator for Cu(ii) from CuCl_2_, likely due to its higher degree of conjugation compared to the other chelators. This suggests that the charged diketone was the most effective for chelating to Cu(ii), forming a square planar complex (ArgusLab prediction, Fig. 5B). In contrast, NO_3_^2−^ and SO_4_^2−^ are weak inner-sphere ligands making their extraction from water difficult due to their high hydration energy.^30^

**Table 2:** The amount of metal extracted from each metal solution by each chelator at a M/L ratio of 10 : 1. pH was not adjusted for these experiments

Entry	Metal salt	Amount of metal extracted/mg L^−1^					
		1	2	3	4	5	6
1	Cu(OAc)_2_	118	84	16	29	232	226
2	Cu(NO_3_)_2_	6	6	0	0	28	78
3	CuSO_4_	5	5	0	0	15	16
4	CuCl_2_	120	108	107	102	88	86
5	CoCl_2_	251	275	279	251	223	181
6	NiCl_2_	0	112	125	0	80	140
7	FeCl_3_	0	81	0	0	167	Emulsion
8	CrCl_3_	0	6	0	0	Emulsion	Emulsion
9	CrCl_3_ (pH = 5–6)^a^	32	238	255	305	Emulsion	Emulsion

a pH was adjusted in this case.

Next, the ability of the chelators to remove different metals, Cr, Fe, Co and Ni, from their corresponding metal chloride solutions was tested, and the results are shown in Table 2 (entries 5–8). It was found that Co(ii) could be removed by all chelators, with 2, 3 and 4 being the most effective, removing 275, 279 and 251 mg L^−1^ Co(ii) respectively. Ni was extracted from a NiCl_2_ solution at pH = 7.27 by 3 (125 mg L^−1^), 5 (80 mg L^−1^) and 6 (140 mg L^−1^) but not with 4 or the traditional chelator 1. This suggests that the neutral diketone group was more involved in the extraction compared to the acetate groups, forming octahedral complexes of the form [M(diketone)_2_Cl_2_], like for Cu(ii), and facilitated extraction to the organic layer. This again demonstrates the enhanced chelating ability of the new bio-derived molecules. Co(ii) removal from a solution of Co(NO_3_)_2_ solution was also attempted but no extraction was observed by any of the chelators. Like in the case of Cu(NO_3_)_2_ this was likely due to NO_3_^2−^ being a weak inner sphere ligand.

Although trivalent metals such as Fe(iii) and Cr(iii) can be extracted using β-diketones, pH adjustment is required. The high charge density of Fe(iii) and Cr(iii) can polarize O–H bonds in water and liberate H^+^ as shown in eqn (1). Consequently, extraction from FeCl_3_ and CrCl_3_ solutions using chelators is generally challenging.

1 [M(H_2_O)_6_]^3+^_(aq.)_ ⇌ [M(H_2_O)_5_OH]^2+^_(aq.)_ + H^+^_(aq.)_

However, the bio-derived chelators show improved extraction ability compared to the commercially available lipophilic chelator dibenzoylmethane 1, as both the unmodified bio-derived diketone 2 and the bifunctional diketone 4 could extract large amounts of Fe(iii) (81 and 167 mg L^−1^ respectively, Table 2). Only very small amounts of Cr(iii) were extracted using unmodified bio-derived diketone 2 (6 mg L^−1^). By increasing pH in a CrCl_3_ solution to 5–6, a significant improvement in extraction performance was observed (Table 2, entry 9). Superior levels of extraction were observed with the bio-derived chelators 2, 3 and 4 (238, 255 and 305 mg L^−1^ respectively) compared to 1 (32 mg L^−1^). This result would hint towards potential positive benefit to the chelation ability of the diketone by attaching neighbouring ester groups that are assumed to not normally directly bind to the metal ion. An octahedral complex is known to form with acetylacetonate, and a similar complex is believed to have formed using the chelators in this work (ArgusLab prediction, Fig. 5C).

While interaction between the bio-derived chelators and Cr(iii) was apparent, no phase separation was observed when using 5 and 6 for the extraction of Cr(iii). The same problem was observed when using 6 for the extraction of Fe(iii). An emulsion may have formed due to the polarized acid groups resulting from the high charge density of Fe(iii) and Cr(iii) on the otherwise lipophilic β-diketone. This observation would hint towards potential applications of 5 and 6 as surface active agents, whilst also highlighting the merit of using the less-polar ester derivatives as chelators to facilitate separation of the biphasic system.

A competitive extraction was carried out using a solution containing an equal concentration of CuCl_2_ and CoCl_2_ (15 mM) at pH = 5, as can be seen in Table 3. Like in the single metal chloride solutions, 3, 4, 5 and 6 removed more cobalt than copper. 1 was marginally more selective for copper than cobalt in the mixed metal chloride solution, in contrast to its performance in single metal chloride solutions. Interestingly, chelator 4 was completely selective for cobalt in the mixed solution, which opens up the possibility for applications in metal separation. The competitive extraction of Cu and Co with non-coordinating anions was not undertaken in this study and would be a priority for future work.

**Table 3:** Competitive extraction of Cu and Co from a solution containing their corresponding chloride salts

Chelator	Amount of metal extraction/mg L^−1^	
	Co	Cu
1	59	64
2	87	64
3	59	32
4	30	0
5	91	29
6	73	38

## Conclusions

In conclusion, the use of bio-platform molecules, heterogeneous catalysis and microwave heating in the absence of a solvent results in a green process which yields a range of bio-derived lipophilic bifunctional “super-chelators” with enhanced chelation ability compared to both commercially available and unmodified β-diketones chelators (1 and 2). Importantly, the modification of 2 by the Michael addition reaction with bio-derived acids enhances the chelating ability of the β-diketone. In many cases, the new chelators significantly outperformed the commercially available hydrophobic chelator, 1. For example, 232 and 226 mg L^−1^ Cu(ii) was extracted from Cu(OAc)_2_ using 5 and 6 respectively compared to 118 mg L^−1^ by 1, due to the extra carboxylate functionality on the new chelators. Critically, the presence of bifunctional groups leads to an enhancement over the unmodified β-diketone 2, for the chelation of both Fe(iii) and Cr(iii). The modified β-diketone 5 was able to extract 167 mg L^−1^ of Fe(iii) from an FeCl_3_ solution with no pH adjustment. No chelation was observed for the commercial chelator 1, while the unmodified β-diketone 2 was able to extract 81 mg L^−1^. The modified super-chelators 3 and 4 extracted 255 and 305 mg L^−1^ Cr(iii) from a solution of CrCl_3_ at pH 5–6, whilst no extraction was observed using 1 and the unmodified β-diketone 2 was able to extract 238 mg L^−1^. It was also found that at higher pH almost quantitative extraction was achieved using the super-chelators. Further investigation into the new chelators is required, such as toxicity and biodegradability testing, whilst promise is also indicated for their use as novel surfactants.

## Conflicts of interest

There are no conflicts of interest to declare.

## Supplementary Material

RA-009-C8RA09426H-s001
