# Supplementary material for: Modification of bio-based β-diketone from wheat straw wax: synthesis of polydentate lipophilic super-chelators for enhanced metal recovery
Source: RSC Adv. 2019 Jan 25;9(7):3542–9. doi: 10.1039/c8ra09426h (PMC9060256; doi:10.1039/c8ra09426h)
Supplement: RA-009-C8RA09426H-s001 [file RA-009-C8RA09426H-s001.pdf]

Electronic Supplementary Material (ESI) for RSC Advances.
This journal is © The Royal Society of Chemistry 2019

Electronic Supplementary Material (ESI) for Chemical Communications
This journal is © The Royal Society of Chemistry

Electronic Supporting Information

Modification of bio-based β-diketone from wheat straw wax: Synthesis of polydentate lipophilic super-chelators for enhanced metal recovery

Kaana Asemave,[a,b] Fergal P. Byrne,[a] James H. Clark,[a] Thomas J. Farmer*[a] and Andrew J. Hunt*[c]

[a.] Green Chemistry Centre of Excellence, University of York, York YO10 5DD, UK.
[b.] Chemistry Department, Benue State University, Makurdi, Nigeria.
[c.] Materials Chemistry Research Center, Department of Chemistry and Center of Excellence for Innovation in Chemistry, Faculty of Science, Khon Kaen University, Khon Kaen, 40002, Thailand.

Corresponding authors email: thomas.farmer@york.ac.uk and andrew@KKU.ac.th

Content:

S1. Additional Experimental Section S2

S1.1. Materials and equipment S2

S1.2. Purification and characterisation of hentriacontane-14,16-dione from wheat straw wax S2

S1.3. Preparation of KF/alumina catalyst S3

S1.4. Preparation and characterisation of trimethyl aconitate S3

S1.5. Modification and characterisation of bio-derived β-diketone S3

S1.6. Hydrolysis of the esters of modified β-diketone with subsequent characterisation S4

S1.7. Procedure the extraction of metal ions S5

S1.8. Prediction of Structure of Metal-chelator Complexes Using Argus Lab Software S5

1. Additional Experimental Section

1.1. Materials and equipment

Cyclohexane, ethyl acetate, hydrochloric acid, petroleum ether (60–80 °C), potassium hydroxide and sodium hydroxide were purchased from Fisher Scientific UK Limited, TLC plates were supplied by Merck, deuterated chloroform, methyl acrylate, dimethyl itaconate, methyl methacrylate, potassium fluoride, alumina and methyl *trans*-cinnamate were purchased from Sigma-Aldrich, all with purity of 99%. Wheat straw wax was extracted by supercritical CO_2 using a Thar SFE-500 extractor at the Green Chemistry Centre of Excellence at the University of York. Extractions were conducted using a previously published method and conditions.[6]

GC-MS was carried out on a Perkin Elmer Clarus 500 GC coupled with a Clarus 500 quadrupole mass spectrometer; GC-FID was carried out on an Agilent 6890N, fitted with an RXI-5HT column (400 ^{O}C column maximum temperature) with dimensions 30 m x 0.25 mm x 0.25 µm; Infra-red spectrometry was carried out on an FTIR Bruker Vertex 70 machine; ^{1}H-NMR and ^{13}C-NMR was carried out on a JEOL JNM-ECS 400 MHz spectrometer. 16 scans were used for ^{1}H NMR analysis, and 256 scans were used for ^{13}C NMR analysis. NMR data were processed and analysed by ACD/NMR Processor Academic Edition software (Ver. 12.01); Differential canning calorimetry was carried out on a MDSC Q2000 machine; a CEM discover microwave reactor was used for microwave heated reactions; LC-MS was carried out on an Agilent 1260 Infinity and Bruker micro-TOF time of flight MS.

1.2. Purification and characterisation of hentriacontane-14,16-dione from wheat straw wax

The isolation of the hentriacontane-14,16-dione (HTD) was carried out as previously reported by Horn *et al.*[21] with some slight modifications. A typical extraction procedure is as follows: Wheat straw wax (1 g) was thoroughly crushed with spatula in a 500 mL beaker and dissolved with petroleum ether (60- 80 °C) (200 mL) at room temperature with magnetic stirring. This mixture was filtered after standing for 30 mins and the filtrate transferred to 500 mL separating flask, along with hot excess saturated aqueous $Cu(OAc)_2$ (100 mL). The resulting mixture was shaken intermittently for 10 mins and allowed to stand for 1 h. The organic phase (upper layer) became green, while the aqueous phase (lower layer) was pale blue. A dark blue precipitate (Cu-diketone) formed upon cooling, so the system was kept warm with a heat gun to allow separation of the organic and aqueous phase. The aqueous layer was removed while the organic layer was collected and kept for 2 hrs to allow precipitation of the Cu-diketone. The precipitate was then filtered, and the crude Cu-diketone washed with petroleum ether (60 – 80 °C) (5 mL). The copper salt was re-dissolved in hot petroleum ether (60 – 80 °C) (100 mL), transferred into the 500 mL separating flask followed by concentrated hydrochloric acid (2 mL). The separating flask and its contents were thoroughly shaken intermittently for 10 mins to strip out the Cu(II) ions from the lipophilic *β*-diketone in the organic phase. The organic layer was washed three times with 100 mL deionised distilled water. The two layers were separated and the pale-yellow product (HTD) was recovered from the organic layer by removing the petroleum ether *in vacuo*. The excess aqueous cuprous acetate was recovered and re-used. ^{1}H-NMR (400 MHz, *$CDCl_3$*) δ ppm 0.79 – 0.92 (m, 9H), 1.24 (s, 60H), 1.41 (s, 3H), 1.37 (s, 4H), 1.49 – 1.75 (m, 16H), 2.22 – 2.31 (m, 5H), 2.48 (t, *j* = 732 Hz, 1H), 3.53 (s, 1H), 5.46 (s, 1H). ^{13}C-NMR (101 MHz, *$CDCl_3$*) δ ppm 14.19 (1 C, s), 22.77 (1 C, s), 25.83 (1 C, s), 29.32 (1 C, s), 29.44 (1 C, s), 29.55 (1 C, s), 29.69 (1 C, s), 29.73 (1 C, s), 29.76 (1 C, s), 32.00 (1 C, s), 38.50 (1 C, s), 99.13 (1 C, s), 123.71 (1 C, s), 193.28 (1 C, s), 194.66 (1 C, s). FTIR (cm^{-1}) - 2955, 2916, 2849, 1639, 1453, 1419, 1375, 1139, 907, 786, 722, 721, 631. GC-MS molecular ion; 464 m/z; Yield 18 ±5wt%. Melting point, 53.9 ^{O}C; r_f

(80% cyclohexane and 20% ethyl acetate): 0.89±0.03, ESI-MS(+), 463.4506 m/z, 503.4437 m/z, 549.4853 m/z.

1.3. Preparation of KF/alumina catalyst

KF/alumina was prepared as previously reported by Farmer *et al.*[19] and Lenardão *et al.*[18] Alumina was pre-treated by drying in oven at 150 °C for 12 hrs. Dried alumina (10 g) was mixed with KF (2.9 g, 50 mmol) and dissolved in methanol (20 mL) in a 250 mL round-bottom flask. This mixture was stirred for 30 mins at 50 °C. Thereafter, methanol was removed *in vacuo* with mixing.[19] The KF/alumina was dried in a vacuum oven for 14 hrs at 150 °C. This mixture produced 5 mmol KF/g alumina.

1.4. Preparation and characterisation of trimethyl aconitate

Aconitic acid (5 g, 0.029 mol) and methanol (33.3 mL, 0.823 mol, 30 molar equivalents) were placed into 250 mL round bottom flask along with 3 drops of concentrated sulphuric acid. The reaction mixture was refluxed overnight with continuous stirring. After 18 hrs, the methanol was removed and fresh methanol (33.3 mL, 0.823 mol) and 3 more drops of sulfuric acid were added. The system was refluxed for another 18 h. After the reaction, the reaction mixture was transferred to a separating funnel along with water (75 mL). The reaction vessel was rinsed with petroleum ether (60-80 °C). After shaking, settling and separating, the organic layer was washed with 15 mL of distilled water, followed by 25 mL of 5% sodium bicarbonate, followed by 15 mL brine solution, at which point it was dried with anhydrous magnesium sulphate and filtered. The solvent was removed *in vacuo* to give the product, a pale-yellow oil. The yield obtained was 85%.

^{1}H NMR (400 MHz, *$CDCl_3$*): δ (ppm) 3.62 (3 H, s), 3.70 (3 H, s), 3.75 (3 H, s), 3.89 (2 H, s), 6.78-6.96 (1 H, m); ^{13}C-NMR (100 MHz, *$CDCl_3$*): δ (ppm) 32.85 (1 C, s), 52.05 (1 C, s), 52.17 (1 C, s), 52.89 (1 C, s), 129.03 (1 C, s), 139.86 (1 C, s), 165.71 (1 C, s), 166.37 (1 C, s), 170.30 (1 C, s); *m/z* (%): (ESI-MS) 216 [M].

1.5. Modification and characterisation of bio-derived β-diketone

Based on the method previously reported by Asemave et al.[22] Into a 15 mL microwave vial, 14,16-hentriacontanedione (HTD) (0.010 g, 0.02 mmol) was added; followed by a known amount of KF/alumina. The heterogeneous catalyst was well dispersed among the HTD. Dimethyl itaconate (0.014 g, 0.09 mmol, 4.5 mole equivalents) or methyl acrylate (10 µL, 0.11 mmol, 5.5 mole equivalents) was then added to the reaction mixture for the synthesis of **3** and **4** respectively. The maximum pressure and the power in the CEM discover microwave was set at 300 psi and 300 watts respectively at a known reaction time and temperature. After the reaction, the reaction mixture was filtered to remove the catalyst using dichloromethane and ethyl acetate. The products were purified using flash chromatography. In addition, short part distillation (Kugelrohr) was also applied to separate the excess dimethyl itaconate from the modified HTD.

When the reaction was conducted with conventional heat source (stirrer hot plate), identical quantities of materials were added to a 15 mL vial with screw cap. The reaction mixture was well stirred during the reaction time under solvent-less conditions. The reactions were also scaled up

using 0.2 g HTD, 1 g KF/alumina with 4.5 mole equivalents of the dimethyl itaconate or 5.5 mole equivalents of the methyl acrylate.

[**3**] ^{1}H NMR (400 MHz, *CDCl$_3$*): δ (ppm) 0.76-0.86 (6 H, m), 1.11-1.29 (41 H, m), 1.30-1.38 (3 H, m), 1.40-1.53 (5 H, m), 1.98-2.06 (3 H, m), 2.11-2.18 (4 H, m), 2.27 (4 H, t, *J*=7.32 Hz), 3.60 (6 H, s); ^{13}C NMR (101 MHz, *CDCl$_3$*): δ (ppm) 14.21 (s, 1 C), 22.77 (s, 2 C), 23.67 (s, 1 C), 25.45 (s, 1 C), 28.79 (s, 1 C), 29.17 (s, 1 C), 29.37-29.61 (m, 4 C), 29.62-29.89 (m, 7 C), 31.55 (s, 1 C), 32.00 (s, 2 C), 39.29 (s, 1 C), 41.24 (s, 1 C), 42.27 (s, 1 C), 51.96 (s, 1 C), 68.77 (s, 1 C), 173.12 (s, 1 C), 193.09 (s, 1 C), 208.29 (s, 1 C); FTIR 2915, 2849, 1723, 1689, 1475, 1462, 1438, 1408, 1375, 1258, 1234, 1200, 1175, 1133, 1110, 1067, 985, 823, 728, 718, 653, 627 cm^{-1}; *m/z* (%): (ESI-MS) 675.5 ($\mathbf{3}_d$) [M$^+$], 589.5 ($\mathbf{3}_s$) [M$^+$]; r_f (80% cyclohexane and 20% ethyl acetate): 0.59±0.05 ($\mathbf{3}_s$) and 0.41±0.05 ($\mathbf{3}_d$); m.p. = 37.0 °C (DCS).

[**4**] ^{1}H NMR (400 MHz, *CDCl$_3$*): δ (ppm) 0.84-0.90 (5 H, m), 1.18-1.32 (42 H, m), 1.53 (7 H, s), 1.47-1.57 (3 H, m), 2.05 (2 H, s), 2.36-2.52 (4 H, m), 2.65-2.74 (2 H, m), 3.66 (2 H, s), 3.68 (2 H, s), 3.77 (1 H, dd, *J*=8.70, 5.50 Hz); ^{13}C NMR (101 MHz, *CDCl$_3$*): δ (ppm) 14.21 (s, 1 C) 23.44 (s, 1 C) 29.10 (s, 1 C) 29.22-29.86 (m, 10 C) 32.01 (s, 3 C) 36.41 (s, 1 C) 39.45 (s, 1 C) 41.85 (s, 1 C) 42.67 (s, 1 C) 52.01 (s, 1 C) 52.19 (s, 1 C) 65.12 (s, 1 C) 171.80 (s, 1 C) 205.54 (s, 1 C); FTIR 2916, 2850, 1735, 1729, 1717, 1692, 1583, 1543, 1471, 1440, 1373, 1286, 1223, 1168, 1106, 1011, 967, 888, 844, 718, 630, 544, 537, 530, 521, 515, 503 cm^{-1}; ESI-MS; (m/z) 661.5 [M$^+$]; r_f (80% cyclohexane and 20% ethyl acetate): 0.51±0.05; m.p. = 46.7 °C (DSC).

1.6. Hydrolysis of the esters of modified β-diketone with subsequent characterisation

3 (0.0692 g) and **4** (0.0479 g) were dissolved separately in dichloromethane in 50 mL vials. Into these solutions was added a saturated solution of sodium hydroxide in methanol. The ratio of dichloromethane:sodium hydroxide solution was 1.5:1. The two mixtures were stirred overnight at 30 °C. The mixtures were dark yellow at the beginning of the reaction and became white and cloudy over time, indicating formation of the Na salts. The reactions were stopped, and the solvent removed. The solid residues were then dissolved separately in 10 mL water and acidified with hydrochloric acid to a pH of 1-2, in order to obtain the carboxylic acid forms of the products (**5** and **6**). The mixtures were then extracted using dichloromethane.

[**5**] ^{1}H NMR (400 MHz, *CDCl$_3$*): δ (ppm) 0.86 (7 H, s), 1.24 (47 H, s), 1.42 (3 H, br. s.), 1.54 (2 H, br. s.), 1.61 (2 H, br. s.), 1.88 (1 H, s), 2.33 (4 H, s), 2.48 (2 H, s); ^{13}C-NMR (101 MHz, *CDCl$_3$*) δ ppm 14.23 (s, 3 C) 22.79 (s, 3 C) 23.61 (s, 1 C) 24.77 (s, 1 C) 25.26 (s, 1 C) 29.46 (s, 2 C) 29.79 (br. s., 4 C) 32.02 (s, 2 C) 33.95 (s, 1 C) 42.69 (s, 1 C) 132.28 (s, 1 C) 165.90 (s, 1 C) 179.01 (s, 1 C) 207.62 (s, 1 C); FTIR 3400-2400 (broad), 2916, 2849, 1700, 1472, 1464, 1431, 1291, 1250, 1228, 1210, 1198, 1094, 933 (–OH bend), 728, 720, 688, 544, 529, 518, 512 cm^{-1}; ESI-MS; 647 *m/z* [M$^+$].

[**6**] ^{1}H NMR (400 MHz, *CDCl$_3$*) δ (ppm) 0.77-0.90 (m, 9 H), 1.12-1.35 (m, 46 H), 1.62 (s, 3 H), 1.88 (s, 1 H), 2.29-2.43 (m, 4 H), 2.54 (d, *J*=7.33 Hz, 2 H), 2.76 (d, *J*=16.49 Hz, 1 H), 2.83 (br. s., 1 H); ^{13}C NMR (101 MHz, *CDCl$_3$*): δ (ppm) 14.23 (s, 3 C), 22.79 (s, 4 C), 23.34-25.31 (m, 2 C), 28.48-29.94 (m, 8 C), 32.02 (s, 3 C), 34.04 (s, 1 C), 39.39-40.53 (m, 1 C), 43.09 (s, 1 C), 129.07-130.98 (m, 2 C), 166.30 (s, 1 C), 178.95-180.86 (m, 1 C), 210.18 (s, 1 C); FTIR 3400- 2500, 2916, 2849, 1694, 1433, 1380, 1283, 1236, 942, 720 cm^{-1}. ESI-MS, 383.2 m/z [M$^-$], 355.2 m/z [M$^-$].

1.7. Procedure the extraction of metal ions

An equal volume (5 mL) of an aqueous metal solution of known molarity was combined with a cyclohexane/chelator solution of known molarity in a 50 mL vial with screw cap at 20 °C and vigorously agitated for 30 mins, as previously reported in the literature.[7,23,24] Thereafter, the sample was allowed to stand for 24 h. A blank (control) sample containing an aqueous metal solution and cyclohexane only was treated as described above to determine the distribution ratio, *D*. The aqueous phase was carefully removed and the absorbance of the residual metal ions in the aqueous solution was measured by UV/Visible spectrophotometer. The concentration of metal in the raffinates and blanks were measured by carefully removing the aqueous phase from the mixtures and measuring the absorbance of the residual metal ions by UV/Visible spectrophotometer. The UV/Visible spectrophotometer was calibrated using aqueous metal solutions of varying concentrations. The actual amount of extracted metal was determined by multiplying the observed amount by the distribution ratio, *D*.[25,26] The pH of the aqueous phase after extraction was recorded as the equilibrium pH. The pH was adjusted with sodium hydroxide or hydrochloric acid in some cases as specified.

Table S1. The changes in pH of each metal solution after extraction with each chelator at a M/L ratio of 10:1.

			pH at the point of extraction					
Entry	Metal salt	Initial pH	**1**	**2**	**3**	**4**	**5**	**6**
1	$Cu(OAc)_2$	6.13	5.85	5.84	5.96	5.95	5.48	5.73
2	$Cu(NO_3)_2$	5.00	2.93	3.95	-	-	4.22	3.75
3	$CuSO_4$	4.84	3.04	4.19	-	-	3.89	3.91
4	$CuCl_2$	4.17	2.48	3.72	4.61	4.59	3.37	3.05
5	$CoCl_2$	7.29	7.20	6.98	7.38	7.38	6.52	5.50
6	$NiCl_2$	7.27	-	6.82	7.29	-	5.50	6.73
7	$FeCl_3$	1.59	-	1.52	-	-	1.42	Emulsion
8	$CrCl_3$	2.58	-	2.57	-	-	Emulsion	Emulsion

(-) indicates no change due to no metal extraction observed, as can be seen in Table 3.

1.8. Prediction of Structure of Metal-chelator Complexes Using ArgusLab Software

In this work, ArgusLab (obtainable at http://www.arguslab.com/arguslab.com/ArgusLab.html) was used to optimise (using the Austin Model 1 (AM1) and a restricted Hartree-Fock (RHF) calculation) and produce images of the predicted molecular geometry for a selection of the chelators.
